# Proximal fibular osteotomy versus high tibial osteotomy for treating knee osteoarthritis: a systematic review and meta-analysis

**DOI:** 10.1186/s13018-022-03299-8

**Published:** 2022-10-28

**Authors:** Zhan-Xiong Wu, Wen-Xia Ren, Zhi-Qiang Wang

**Affiliations:** Department of Orthopedics, Shanxi Bethune Hospital and Shanxi Academy of Medical Sciences, No.99, Longcheng street, Xiaodian District, Taiyuan, 030009 Shanxi Province People’s Republic of China

**Keywords:** Osteoarthritis, Tibial plateau, Fibular, Osteotomy, Systematic review, Meta-analysis

## Abstract

**Background:**

Knee osteoarthritis (KOA) with varus alignment and medial space stenosis is a common degenerative disorder in the elderly. To reallocate the force bearing from the medial to the lateral compartment, the anti-varus osteotomy, including high tibial osteotomy (HTO) and proximal fibular osteotomy (PFO), corrects the mechanical lines of lower extremities using surgical methods, which alleviates the abrasion of medial cartilage and relieves pain. PFO is based on the “non-uniform settlement” theory. It is to cut small section of the proximal fibula, i.e., below the fibula head, which breaks the fibula and weakens its support for the lateral of the tibial plateau, lastly reduces the gap on the lateral side of the knee joint and offsets the knee varus deformity caused by weight bearing. We conducted this systematic review and meta-analysis to compare the clinical outcomes of PFO versus HTO intervention.

**Methods:**

Twenty-three studies were acquired from PubMed, Embase, CNKI (China National Knowledge Infrastructure), Wanfang Database and Cochrane Library. The data were extracted by two of the coauthors independently and were analyzed by RevMan5.3. Mean differences (MDs), odds ratios (ORs) and 95% confidence intervals (CIs) were calculated. Cochrane Collaboration’s Risk of Bias Tool and Newcastle–Ottawa Scale were used to assess risk of bias.

**Results:**

Twenty-three studies including 14 randomized controlled trials and 9 observational studies were assessed. The methodological quality of the trials ranged from low to high. The pooled results of the mean operation time (MD =  − 38.75, 95% CI =  − 45.66 to − 31.85, *P* < 0.00001), intraoperative bleeding (std. MD =  − 4.12, 95% CI =  − 5 to − 3.24, *P* < 0.00001), length of hospital stay (MD =  − 3.77, 95% CI =  − 4.98 to − 2.56, *P* < 0.00001) and postoperative complications (OR = 0.66, 95% CI = 0.37–1.18, *P* = 0.16) showed that the differences were statistically significant between the two interventions. The postoperative differences of visual analogue score (VAS) (MD = 0.15 95% CI =  − 0.39 to 0.69, *P* = 0.58), hospital for Special Surgery knee score (HSS) (MD =  − 2.68, 95% CI =  − 6.30 to 0.94, *P* = 0.15), American knee society (AKS) score (MD = 0.04, 95% CI =  − 0.69 to 0.77, *P* = 0.91), western Ontario and McMaster university of orthopedic index (WOMAC) (MD = 8.09, 95% CI = 2.06–14.13, *P* = 0.009) and femur–tibia angle (FTA) (MD =  − 0.03, 95% CI =  − 5.39 to 5.33, *P* = 0.99) were not statistically significant. Sensitivity analysis proved the stability of the pooled results and the publication bias was not apparent.

**Conclusions:**

PFO and HTO have the same short-term efficacy in the treatment of KOA, but PFO can reduce the operation time, intraoperative bleeding, hospital stay and postoperative complications, which has certain advantages. Clinically, for patients with many complications and poor surgical tolerance, PFO can be preferred.

## Introduction

Knee osteoarthritis (KOA) is a common degenerative disease, mainly characterized by slow progressive pain, swelling, stiffness and dysfunction of the knee joint [1]. A study that included individuals above 60 years of age in the USA has estimated the prevalence of radiographic changes consistent with KOA of the knee to be 37% [2]. At present, knee osteoarthritis cannot be completely cured. With the progression of the disease to the late stage, it has a great impact on the quality of life of patients. At this time, surgery is an effective treatment. Joint replacement is the main scheme for the treatment of severe KOA in the past. Although it can effectively reduce patients' pain and improve their joint function, it has the disadvantages of complex operation, high cost and need to be repaired again. In recent years, with the proposal of “knee preservation concept,” osteotomy is more and more widely used in the treatment of KOA, mainly including high tibial osteotomy (HTO) and proximal fibular osteotomy (PFO) and unicompartmental knee arthroplasty [3–5]. After a long time of development, HTO is convenient and effective, and 10-year survival rates were 91.6% in open wedge HTO [6, 7]. Revision surgery is a challenge that can occur with osteotomies, which could be finally solved by conversion of HTO to total knee arthroplasty (TKA). Patients who undergo conversion of HTO to TKA have similar 10-year survival rate as patients who undergo primary TKA [8]. Compared to other options, PFO is a surgical method which is a simple, easy-to-do and less invasive procedure, which requires only a small incision, limited dissection and no internal fixation [9]. Based on the "theory of uneven settlement of knee joint" [10, 11], they believe that PFO can improve the pressure of medial compartment and effectively delay the development of KOA. This theory is recognized by many scholars. It is to cut small section of the proximal fibula, i.e., below the fibula head, which breaks the fibula and weakens its support for the lateral of the tibial plateau. As such, the muscle attached to proximal fibula, in the situation of the weight bearing, can pull the fibular head along the distal direction, and the tension is transmitted to the lateral femoral condyle. Eventually, the gap on the lateral side of the knee joint is reduced to offset the knee varus deformity caused by weight bearing. In recent years, there are more and more clinical reports on the treatment of KOA by PFO [12, 13]. However, PFO as a new concept of knee preserving osteotomy is widely used in recent decades, and its efficacy and adverse reactions have not been widely verified in clinical practice. HTO has been proved to be effective for a long time, but it has large surgical trauma and slow postoperative recovery, and will increase the probability of tibial plateau fracture and proximal nonunion of fracture. It is not suitable for the elderly or patients with severe osteoporosis [14].

Due to the different advantages and disadvantages of the above two osteotomies, there are some disputes about the choice of the two methods in clinic. This paper uses meta-analysis method to compare the clinical efficacy and safety of PFO and HTO in the treatment of KOA, in order to provide reference for the choice of clinical treatment strategy.

## Materials and methods

Ethical approval or patient consent was not required since the present study was a review of previous published literatures.

### Inclusive criteria of published studies

#### Types of studies

We considered all published and unpublished studies about PFO versus HTO for treating knee osteoarthritis, covering RCTs and observational studies including retrospective and prospective studies. The studies in English and Chinese language were all included.

#### Types of participants

The subjects were patients of KOA with clear diagnostic criteria and surgical indications. All patients had been diagnosed as patients of age greater than 18 years with radiographic evidence of isolated medial knee joint osteoarthritis who had failed conservative measures and were ready to accept osteotomy. Exclusion criteria were multicompartmental arthritis; history of inflammatory arthritis; or history of prior surgery (aside from ligamentous repair) of the knee joint, distal femur or proximal tibia.

#### Types of interventions

The operation method of the experimental group was PFO, and the operation method of the control group was HTO, which were considered. The exclusion criteria were as follows: (1) insufficient clinical outcome data in studies and (2) reviews, letters or conference articles.

#### Types of outcome measures

The primary outcome measures were the clinical outcomes synthesizing the mean operation time, intraoperative bleeding, length of hospital stay, visual analogue score (VAS), hospital for Special Surgery knee score (HSS), American knee society (AKS) score, western Ontario and McMaster university of orthopedic index (WOMAC) and femur–tibia angle (FTA). The secondary outcomes included: postoperative complications. Major complications such as hinge fractures, neurovascular injury, deep infections, nonunion, knee instability, lower extremity deep venous thrombosis, pulmonary embolism and recurrence deformity, and minor complications such as superficial peroneal nerve traction injury, superficial infections, patella baja, numbness and delayed healing were all included.

### Search methods for identification of studies

Five databases [PubMed, Embase, CNKI (China National Knowledge Infrastructure), Wanfang Database and Cochrane Library] were searched using the keywords such as “Osteoarthritis, Knee/knee osteoarthritis/KOA,” “proximal fibular osteotomy/PFO,” “high tibial osteotomy/HTO” through November 2021 to collect relevant studies about the clinical comparisons of DFO versus HTO for treating KOA. The titles and abstracts of potential related articles identified by the electronic search were reviewed. References from retrieved articles were also assessed to extend the search strategy.

### Data collection and quality assessment

Two partners (ZXW and WXR) independently assessed the titles and abstracts of all the studies screened during initial search, and they excluded any clearly irrelevant studies using the inclusion criteria. Data were independently extracted using a standard data form for the first author’s name, year of publication, sample size, gender, age, intervention, country, study design, follow-up and relevant outcome. A third partner (ZQW) would handle any disagreement about inclusion of a study and reach a consensus. Cochrane Collaboration’s Risk of Bias Tool [15] was manipulated for the appraisal of RCT study quality. Observational studies were assessed by the Newcastle–Ottawa Scale including 8 items [16]. A higher overall score indicates a lower risk of bias and a score of 5 or less (out of 9) corresponds to a high risk of bias.

### Statistical analysis

RevMan statistical software v5.3 was used for the meta-analysis. The analysis of continuous variables was conducted by mean difference (MD) and 95% confidence interval (CI). For a dichotomous outcome, we calculated the odds ratios (ORs) and 95% CIs. Heterogeneity was assessed by Chi-squared and *I*^2^. A *P* < 0.05, *I*^2^ > 50% was considered significantly heterogeneous, and random-effect models were applied. Otherwise, fixed-effect models if there was no significant heterogeneity (*P* ≥ 0.05, *I*^2^ ≤ 50%). Sensitivity analysis was performed by omitting one study at a time to determine the stability of pooled results. Publication bias was determined by a funnel plot.

## Results

### Studies identification and inclusion

**Fig. 1:**
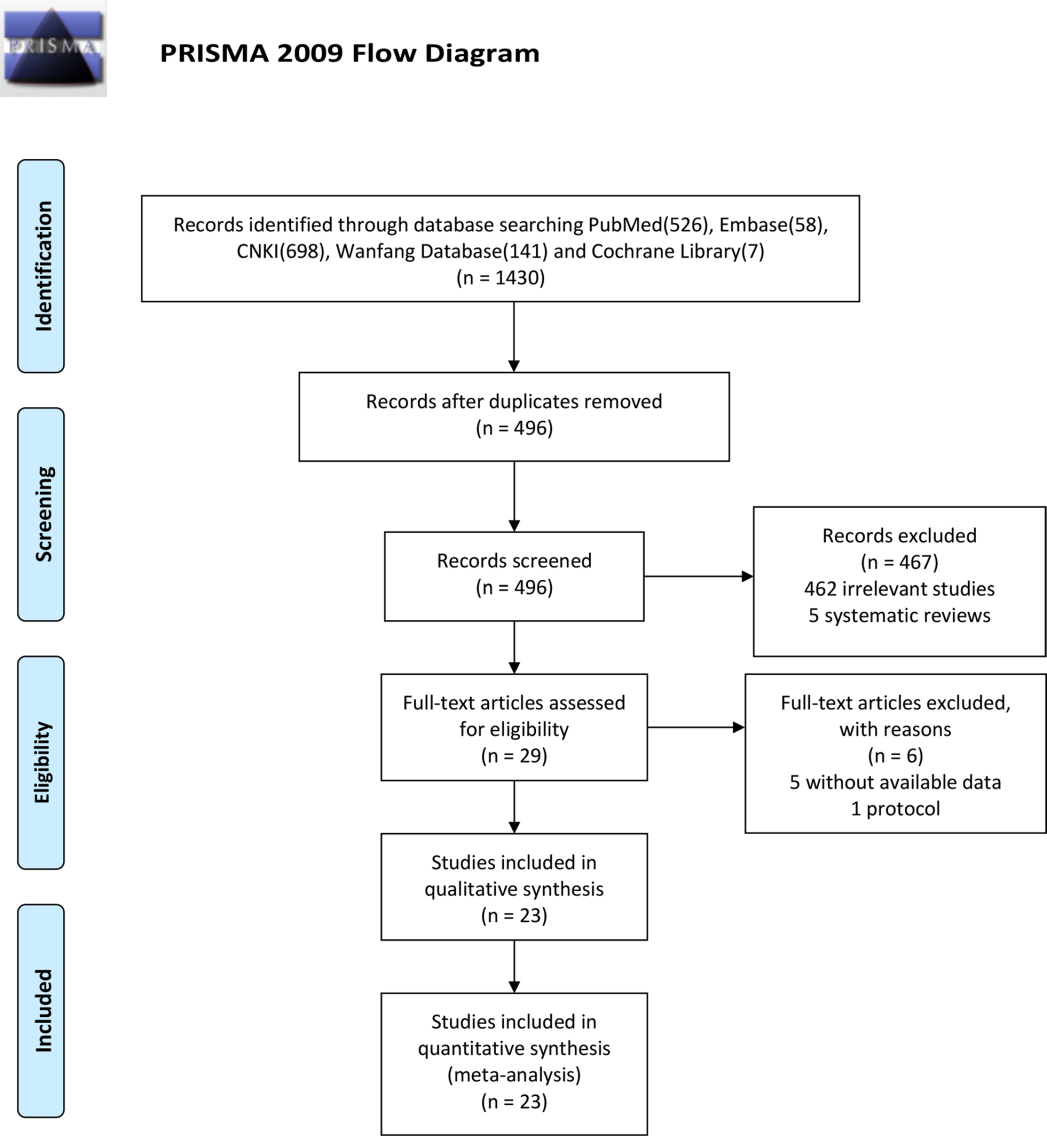
PRISMA flow diagram

Searches conducted in the PubMed, Embase, CNKI, Wanfang Database and Cochrane Library yielded a total of 1430 articles. After removing duplicates, 496 literatures were remained. Based on the titles and abstracts review, 462 irrelevant articles and 5 systematic reviews of them were excluded. Twenty-nine full-text articles were assessed for eligibility. However, six articles were excluded based on the previously established exclusion criteria (5 without available data and 1 protocol). Finally, 23 studies (14 RCTs and 9 observational studies) were included in this systematic review and meta-analysis. The detail of selection process is listed in Fig. 1.

### Study characteristics

We assessed 23 studies [17–39] including 14 RCTs and 9 retrospective studies in this article. The included studies were conducted in 2 countries (Egypt and China) from 2015 to 2021 and involved 1460 patients (710 patients treated with PFO technique, 743 patients treated with HTO technique) aged 48.3–65.6 years. The average follow-up duration ranged from 3 to 24 months. The clinical outcomes of the studies were evaluated mainly based on the mean operation time, intraoperative bleeding, length of hospital stay, VAS, HSS, AKS score, WOMAC, FTA and postoperative complications. The detailed information of included studies is shown in Table 1.

**Table 1:** Characteristics of studies included

	Year	Sample size (PFO/HTO)	Female (%)	Mean age(years)	Intervention PFO HTO		Country	Study design	Follow-up (month)	Relevant outcome
Hu et al. [17]	2021	18/22	60%	PFO 62.84 ± 4.97 HTO 64.05 ± 3.15	Proximal fibular osteotomy	High tibial osteotomy	China	Retrospective study	24	VAS score; HSS
Cai et al. [18]	2020	43/43	40.7%	PFO 61.97 ± 7.95 HTO 62.21 ± 8.58	Proximal fibular osteotomy	High tibial osteotomy	China	Retrospective study	6	Mean operation time; intraoperative bleeding; length of hospital stay; VAS score; HSS; WOMAC; FTA
Mi et al. [19]	2020	21/21	54.8%	66.4 ± 2.5	Proximal fibular osteotomy	High tibial osteotomy	China	RCT study	NR	Mean operation time; intraoperative bleeding; VAS score; HSS
Pian et al. [20]	2020	41/41	70.7%	PFO 59.23 ± 5.89 HTO 58.91 ± 6.10	Proximal fibular osteotomy	High tibial osteotomy	China	RCT study	6	mean operation time; intraoperative bleeding; length of hospital stay; HSS; KSS; postoperative complications
Wang et al. [21]	2019	16/16	53.1%	PFO 53.19 ± 6.62 HTO 52.01 ± 6.01	Proximal fibular osteotomy	High tibial osteotomy	China	RCT study	6	Mean operation time; intraoperative bleeding; length of hospital stay; VAS score; HSS; FTA; AKS; postoperative complications
Ahmed et al. [22]	2019	10/10	40%	PFO 49.27 ± 5.78 HTO 48.30 ± 4.55	Proximal fibular osteotomy	High tibial osteotomy	Egypt	Retrospective study	NR	VAS score; KSS
Li et al. [23]	2019	24/24	43.8%	PFO 55.42 ± 3.17 HTO 54.72 ± 3.96	Proximal fibular osteotomy	High tibial osteotomy	China	RCT study	NR	Mean operation time; intraoperative bleeding; HSS; FTA
Chen et al. [24]	2019	15/15	NR	PFO 60.5 ± 1.4HTO 59.9 ± 1.6	Proximal fibular osteotomy	High tibial osteotomy	China	RCT study	3	Intraoperative bleeding; length of hospital stay; HSS; AKS; postoperative complications
Wang et al. [25]	2019	40/40	65%	PFO 61 ± 5 HTO 56.72 ± 5.99	Proximal fibular osteotomy	High tibial osteotomy	China	Retrospective study	9	mean operation time; intraoperative bleeding; VAS score; HSS; FTA; AKS
Huo et al. [26]	2019	13/13	65.4%	PFO 62.5 ± 1.4 HTO 62.2 ± 1.6	Proximal fibular osteotomy	High tibial osteotomy	China	RCT study	3	Mean operation time; intraoperative bleeding; length of hospital stay; HSS
Ding et al. [27]	2019	49/48	55.7%	PFO 54.43 ± 7.07 HTO 54.02 ± 6.83	Proximal fibular osteotomy	High tibial osteotomy	China	RCT study	6	Mean operation time; intraoperative bleeding; VAS score; HSS
Duan et al. [28]	2019	34/34	45.6%	PFO 71.64 ± 5.24 HTO 71.58 ± 5.21	Proximal fibular osteotomy	High tibial osteotomy	China	RCT study	NR	VAS score; HSS; postoperative complications
Yu et al. [29]	2019	25/25	50%	PFO 54.5 ± 7.2 HTO 53.6 ± 7.2	Proximal fibular osteotomy	High tibial osteotomy	China	Retrospective study	NR	VAS score; HSS; postoperative complications
Qiu et al. [30]	2018	26/37	68.3%	PFO 59 ± 3 HTO 59 ± 3	Proximal fibular osteotomy	High tibial osteotomy	China	Retrospective study	12	mean operation time; intraoperative bleeding; WOMAC; postoperative complications
Zhang et al. [31]	2018	44/44	53.4%	PFO 57.6 ± 2.3 HTO 58.2 ± 2.2	Proximal fibular osteotomy	High tibial osteotomy	China	RCT study	8	Mean operation time; intraoperative bleeding; length of hospital stay; VAS score; AKS
Han et al. [32]	2018	40/40	28.75%	PFO 62.34 ± 10.75 HTO 62.05 ± 10.48	Proximal fibular osteotomy	High tibial osteotomy	China	RCT study	NR	Mean operation time; intraoperative bleeding; length of hospital stay; VAS score; HSS; FTA
Yin et al. [33]	2017	30/30	45%	PFO 62.36 ± 4.53 HTO 63.12 ± 4.33	Proximal fibular osteotomy	High tibial osteotomy	China	RCT study	6	Mean operation time; intraoperative bleeding; length of hospital stay; VAS score; HSS; AKS; postoperative complications
Wang et al. [34]	2017	30/30	51.7%	PFO 56 ± 7 HTO 57 ± 7	Proximal fibular osteotomy	High tibial osteotomy	China	Retrospective study	6	Mean operation time; intraoperative bleeding; VAS score; HSS; FTA; WOMAC; AKS
Chen et al. [35]	2017	73/73	NR	NR	Proximal fibular osteotomy	High tibial osteotomy	China	RCT study	6	Mean operation time; intraoperative bleeding; length of hospital stay; VAS score; HSS
Zou et al. [36]	2017	40/52	70.7%	PFO 62.3 ± 13.5 HTO 65.6 ± 17.2	Proximal fibular osteotomy	High tibial osteotomy	China	RCT study	NR	Mean operation time; intraoperative bleeding; VAS score; postoperative complications
Song et al. [37]	2017	32/33	53.8%	51.3 ± 7.2	Proximal fibular osteotomy	High tibial osteotomy	China	RCT study	12	VAS score; HSS; FTA; WOMAC
Yu et al. [38]	2016	29/27	80.4%	PFO 61 ± 5 HTO 60.72 ± 4.99	Proximal fibular osteotomy	High tibial osteotomy	China	Retrospective study	6.6 ± 1.1	Mean operation time; intraoperative bleeding; length of hospital stay; VAS score; HSS; AKS; postoperative complications
An et al. [39]	2015	24/25	55.1%	PFO 66 ± 8 HTO 64 ± 8	Proximal fibular osteotomy	High tibial osteotomy	China	Retrospective study	12	Postoperative complications

*PFO* proximal fibular osteotomy, *HTO* high tibial osteotomy, *RCT* randomized controlled trial, *VAS* visual analogue scale, *KSS* knee society score, *HSS* hospital for special surgery knee score, *AKS* American knee society score, *WOMAC* western Ontario and McMaster university of orthopedic index, *FTA* femur–tibia angle, *NR*not reported

### Methodological assessment of study quality

**Fig. 2:**
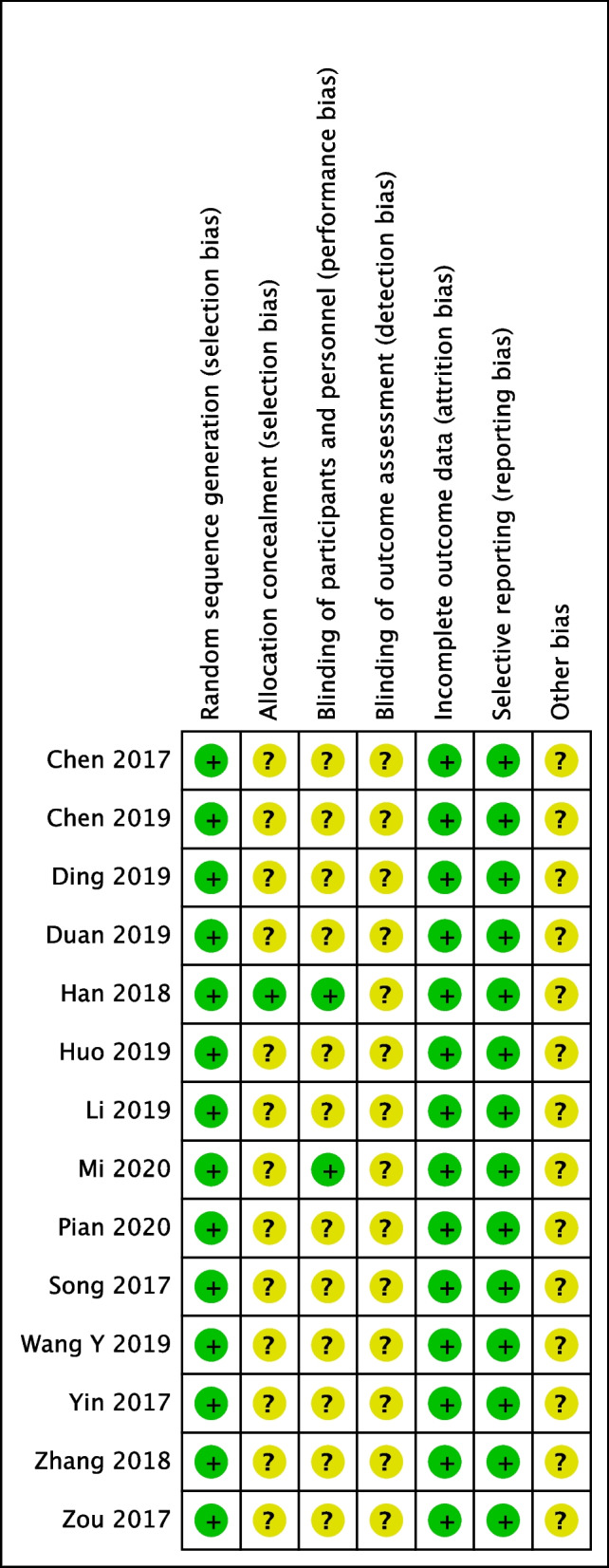
Risk of bias summary: this risk of bias tool incorporates the assessment of randomization (sequence generation and allocation concealment), blinding (participants and outcome assessors), incomplete outcome data, selective outcome reporting and other risk of bias. The items were judged as “low risk,” “unclear risk” or “high risk.” Green means “low risk,” red means “high risk,” and yellow means “unclear risk.”

Methodological quality assessment of the 23 included studies is presented in Fig. 2 and Table 2. Among the RCTs, parts of studies [20, 21, 26, 26, 29, 31, 33, 35, 37] clearly described the random sequence generation by random number tables, but the blinding and allocation concealment were not mentioned, which could be regarded as a low-quality study. Mi’s study [19] was randomly assigned, double-blinded divided, which was considered a moderate-quality study. Han’s study [32] was randomly assigned, double-blinded divided, and the allocation was kept secret with a sealed envelope, which was considered a high-quality study. To sum up, there is no allocation concealment and blind treatment in most RCT literature, and there is a lack of rigorous and careful trial design. Among the observational studies, the Newcastle–Ottawa Scale including the exposed cohort, the non-exposed cohort, ascertainment of exposure, outcome of interest, comparability, assessment of outcome, length of follow-up and adequacy of follow-up, was used to assess the risk of bias. The scores of all 8 studies [17, 18, 22, 25, 29, 34, 38, 39] were all 6–8, indicating a low risk of bias. The score of Qiu’s studies [30] was 5, indicating a high risk of bias.

**Table 2:** Newcastle-Ottawa Scale of observational studies

Study	Selection						Outcome		
	Exposed cohort	Noexposed cohort	Ascertainment of Exposure	Outcome of Interest	Comparability	Assessment of outcome	Length of follow-up	Adequacy of follow-up	Total score
Hu et al. [17]	*	*	*	*	*	*	*	*	8
Cai et al. [18]	*	*	*	*	*	*	*		7
Ahmed et al. [22]	*	*	*	*	*	*			6
Wang et al. [25]	*	*	*	*	*	*	*		7
Qiu et al. [30]	*		*	*		*	*		5
Wang et al. [34]	*	*	*	*	*	*	*		7
Yu et al. [29]	*	*	*	*	*	*	*		7
Yu et al. [38]	*	*	*	*	*	*	*		7
An et al. [39]	*	*	*	*	*	*	*	*	8

*Risk of bias was assessed using the Newcastle–Ottawa Scale. A higher overall score indicates a lower risk of bias; a score of 5 or less (out of 9) corresponds to a high risk of bias

### Comparison of mean operation time between PFO and HTO

**Fig. 3:**
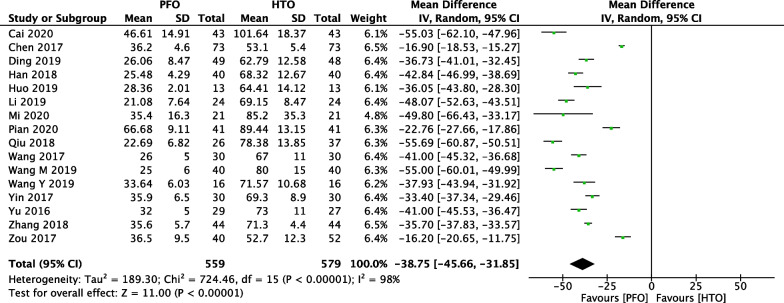
Forest plot of comparison: mean operation time between proximal fibular osteotomy (PFO) and high tibial osteotomy (HTO) for knee osteoarthritis KOA

Comparison of mean operation time between PFO and HTO was conducted among the 16 included studies [18–21, 23, 25–27, 30–36, 38], which included 1138 patients (559 patients receiving PFO and 579 patients receiving HTO), as shown in Fig. 3. Heterogeneity testing showed that there was high heterogeneity among the studies (*P* < 0.00001, *I*^2^ = 98%), so the random-effect model was used to pool the data from the 16 studies. The pooled result showed that the difference was statistically significant between the PFO group and the HTO group (MD =  − 38.75, 95% CI =  − 45.66 to − 31.85, *P* < 0.00001).

### Comparison of intraoperative bleeding between PFO and HTO

**Fig. 4:**
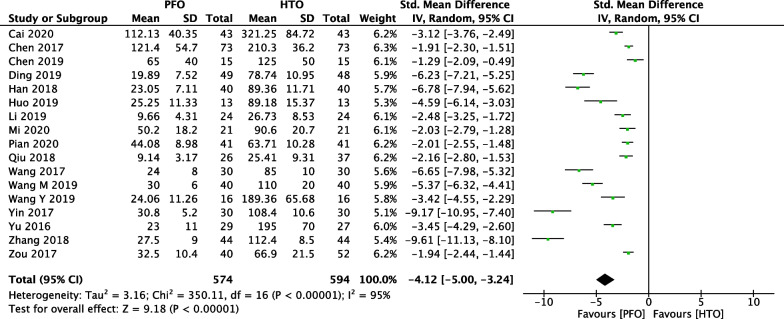
Forest plot of comparison: intraoperative bleeding between proximal fibular osteotomy (PFO) and high tibial osteotomy (HTO) for knee osteoarthritis KOA

In Fig. 4, 17 included studies [18–21, 23–27, 30–36, 38] consisting of 1168 OA patients (574 patients received PFO and 594 patients received HTO technique) reported intraoperative bleeding. High heterogeneity among studies (*P* < 0.00001, *I*^2^ = 95%) was found, so we used the random-effect model. The overall estimate indicated that the pooled Std.MD was − 4.12 (95% CI =  − 5 to − 3.24, *P* < 0.00001,), suggesting that the difference was statistically significant between HTO intervention and PFO intervention.

### Comparison of length of hospital stay between PFO and HTO

**Fig. 5:**
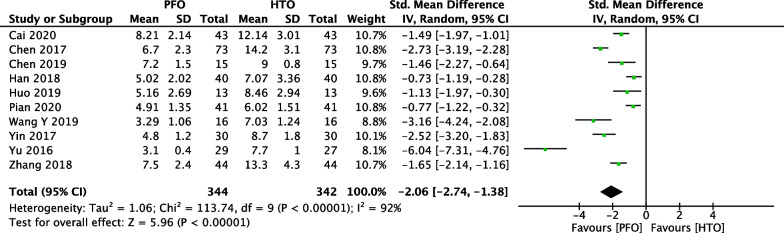
Forest plot of comparison: length of hospital stay between proximal fibular osteotomy (PFO) and high tibial osteotomy (HTO) for knee osteoarthritis KOA

In Fig. 5, ten included studies [18, 20, 21, 24, 26, 31–33, 35, 38] consisting of 686 patients (344 patients received PFO treatment and 342 patients received HTO treatment) investigated length of hospital stay. High heterogeneity among studies (*P* < 0.00001, *I*^2^ = 95%) was found, so we used the random-effect model to pool the data. The overall estimate showed that the difference was statistically significant between the PFO group and the HTO group (MD =  − 3.77, 95% CI =  − 4.98 to − 2.56, *P* < 0.00001).

### Comparison of VAS between PFO and HTO

**Fig. 6:**
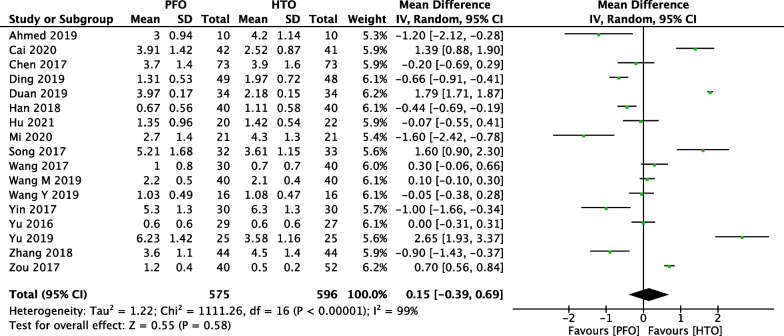
Forest plot of comparison: visual analogue scale (VAS) between proximal fibular osteotomy (PFO) and high tibial osteotomy (HTO) for knee osteoarthritis KOA

Comparison of postoperative VAS score between PFO and HTO treatment was conducted among 17 included studies [17–19, 21, 23, 25, 27–29, 31–38] which contain 1171 patients in Fig. 6. A heterogeneity test showed that there was high heterogeneity among studies (*P* < 0.00001, *I*^2^ = 99%), so the random-effect model was used. The overall estimate showed that the difference between the two groups was not statistically significant (MD = 0.15, 95% CI =  − 0.39 to 0.69, *P* = 0.58).

### Comparison of HSS between PFO and HTO

**Fig. 7:**
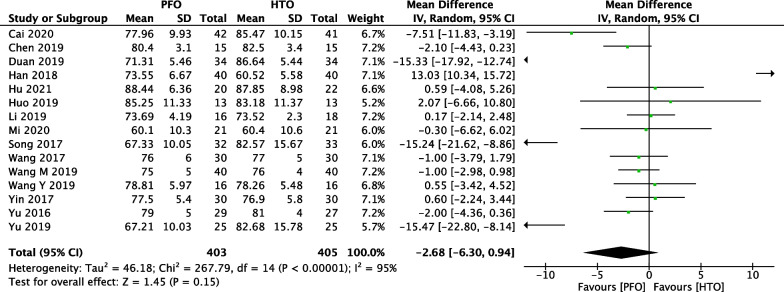
Forest plot of comparison: knee society score (HSS) between proximal fibular osteotomy (PFO) and high tibial osteotomy (HTO) for knee osteoarthritis KOA

In Fig. 7, 15 included studies [17–21, 23–29, 32–35, 37, 38] consisting of 808 patients (403 patients received PFO treatment and 405 patients received HTO treatment) investigated postoperative HSS. High heterogeneity among studies (*P* < 0.00001, *I*^2^ = 95%) was found, so we used the random-effect model to pool the data. The overall estimate showed that the difference was not statistically significant between the PFO group and the HTO group (MD =  − 2.68, 95% CI =  − 6.30 to 0.94, *P* = 0.15).

### Comparison of AKS score between PFO and HTO

**Fig. 8:**
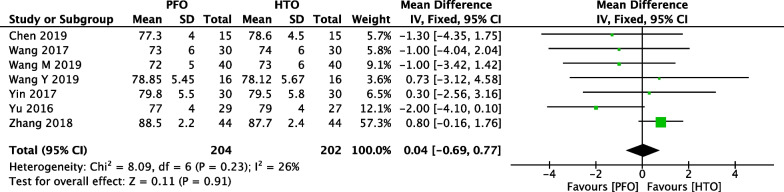
Forest plot of comparison: American knee society score (AKS) between proximal fibular osteotomy (PFO) and high tibial osteotomy (HTO) for knee osteoarthritis KOA

Seven included studies [21, 24, 25, 31, 33, 34, 38] including 204 PFO surgery group cases and 202 HTO surgery group cases provided the data in terms of postoperative AKS score. A heterogeneity test revealed that low heterogeneity existed among the studies (*P* = 0.23, *I*^2^ = 26%) and the fixed-effect model was used. A pooled analysis revealed that there was no significant difference between PFO surgery and HTO surgery group (MD = 0.04, 95% CI =  − 0.69 to 0.77, *P* = 0.91) (Fig. 8).

### Comparison of WOMAC between PFO and HTO

**Fig. 9:**
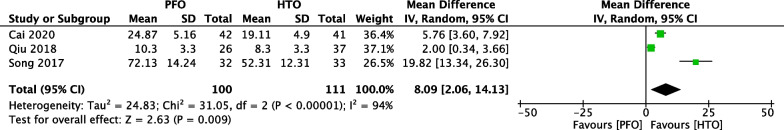
Forest plot of comparison: western Ontario and McMaster University of Orthopedic Index (WOMAC) between proximal fibular osteotomy (PFO) and high tibial osteotomy (HTO) for knee osteoarthritis KOA

Comparison of postoperative WOMAC score between PFO and HTO treatment was conducted among 3 included studies [18, 30, 37] which contain 211 patients in Fig. 9. A heterogeneity test showed that there was high heterogeneity among studies (*P* < 0.00001, *I*^2^ = 94%), so the random-effect model was used. The overall estimate showed that the difference between the two groups was not statistically significant (MD = 8.09, 95% CI = 2.06–14.13, *P* = 0.009).

### Comparison of FTA between PFO and HTO

**Fig. 10:**
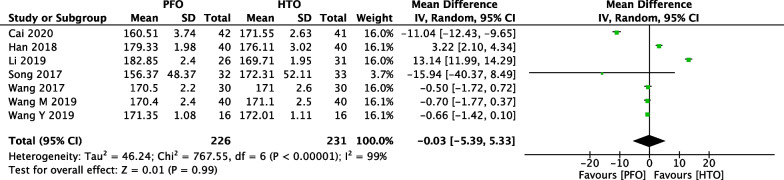
Forest plot of comparison: femur–tibia angle (FTA) between proximal fibular osteotomy (PFO) and high tibial osteotomy (HTO) for knee osteoarthritis KOA

Limb alignment is expressed as the FTA, measuring the lateral angle between the anatomical femoral and tibial axes. the degree of failure was higher when the postoperative FTA was < 5° of anatomical valgus [40]. Comparison of postoperative FTA between PFO and HTO was conducted among the 7 included studies [18, 21, 23, 25, 32, 34, 37], which included 457 patients (226 patients receiving PFO and 231 patients receiving HTO), as shown in Fig. 10. Heterogeneity testing showed that there was high heterogeneity among the studies (*P* < 0.00001, *I*^2^ = 99%), so the random-effect model was used to pool the data from the six studies. The pooled result showed that the difference was not statistically significant between the PFO group and the HTO group (MD =  − 0.03, 95% CI =  − 5.39 to 5.33, *P* = 0.99).

### Comparison of postoperative complications PFO and HTO

**Fig. 11:**
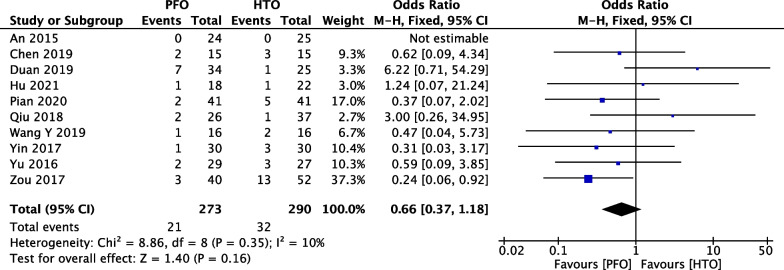
Forest plot of comparison: postoperative complications between proximal fibular osteotomy (PFO) and high tibial osteotomy (HTO) for knee osteoarthritis KOA

In Fig. 11, ten included studies [20, 21, 24, 28–30, 33, 36, 38, 39] consisting of 563 OA patients (273 patients received PFO and 290 patients received HTO technique) reported postoperative complications. Low heterogeneity among studies (*P* = 0.35, *I*^2^ = 10%) was found, so we used the fixed-effect model. The overall estimate indicated that the pooled OR was 0.66 (95% CI = 0.37–1.18, *P* = 0.16), suggesting that the difference was statistically not significant between HTO intervention and PFO intervention.

### Sensitivity analysis and publication bias

**Fig. 12:**
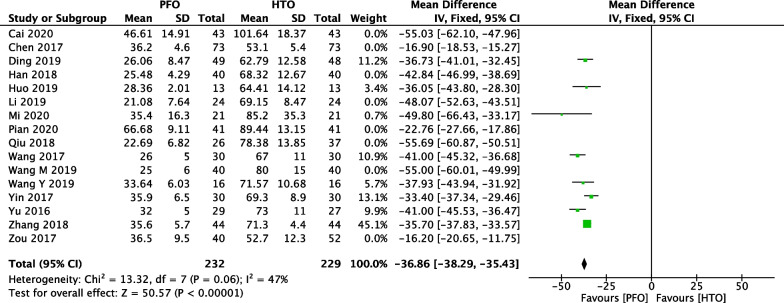
Forest plot of comparison: mean operation time between proximal fibular osteotomy (PFO) and high tibial osteotomy (HTO) for knee osteoarthritis KOA after omitting some low-quality studies by sensitivity analysis

**Fig. 13:**
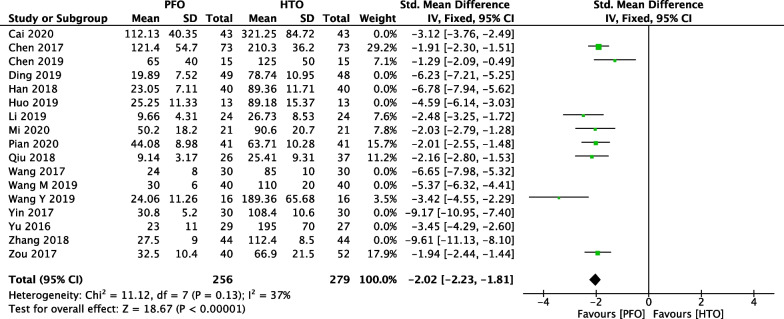
Forest plot of comparison: intraoperative bleeding between proximal fibular osteotomy (PFO) and high tibial osteotomy (HTO) for knee osteoarthritis KOA after omitting some low-quality studies by sensitivity analysis

**Fig. 14:**
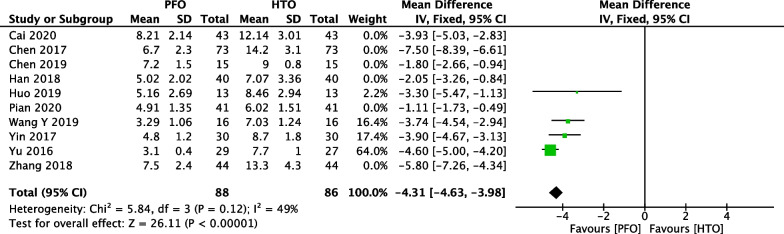
Forest plot of comparison: length of hospital stay between proximal fibular osteotomy (PFO) and high tibial osteotomy (HTO) for knee osteoarthritis KOA after omitting some low-quality studies by sensitivity analysis

**Fig. 15:**
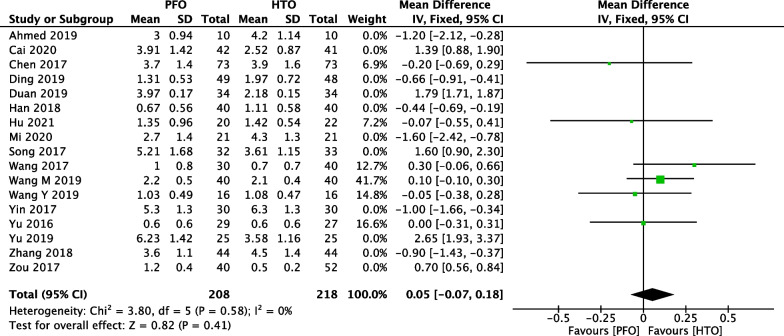
Forest plot of comparison: visual analogue scale (VAS) between proximal fibular osteotomy (PFO) and high tibial osteotomy (HTO) for knee osteoarthritis KOA after omitting some low-quality studies by sensitivity analysis

**Fig. 16:**
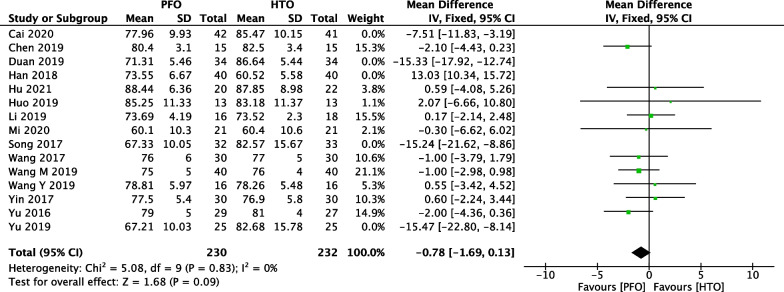
Forest plot of comparison: knee society score (HSS) between proximal fibular osteotomy (PFO) and high tibial osteotomy (HTO) for knee osteoarthritis KOA after omitting some low-quality studies by sensitivity analysis

**Fig. 17:**
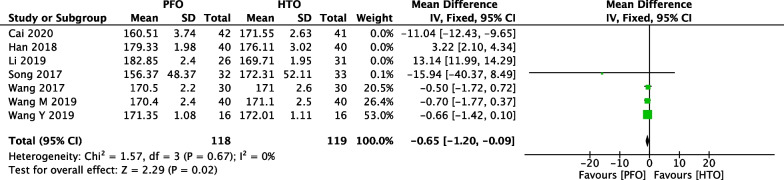
Forest plot of comparison: femur–tibia angle (FTA) between proximal fibular osteotomy (PFO) and high tibial osteotomy (HTO) for knee osteoarthritis KOA after omitting some low-quality studies by sensitivity analysis

**Fig. 18:**
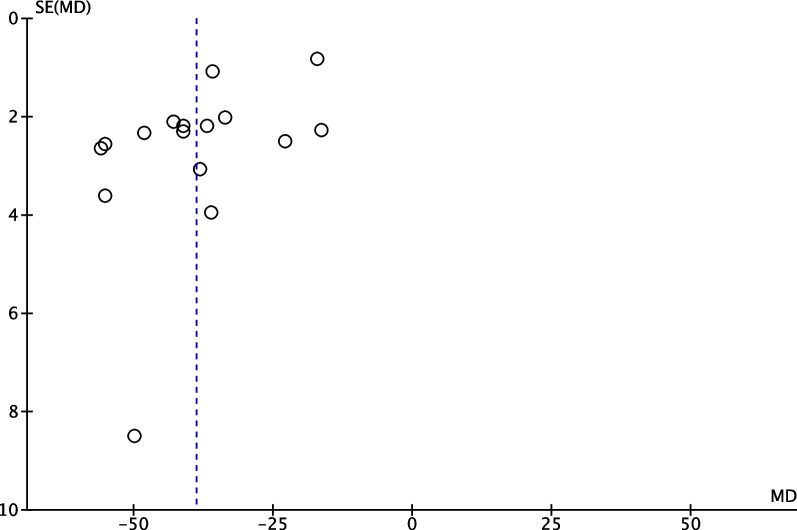
Funnel plot to test for publication bias. Each point represents a separate study for the indicated association. The vertical line represents the mean effects size. MD = mean difference; SE = standard error

We performed a sensitivity analysis to assess the stability of the pooled results. Among the comparisons of operation time (Fig. 12), intraoperative bleeding (Fig. 13), length of hospital stay (Fig. 14), VAS (Fig. 15), HSS (Fig. 16) and FTA (Fig. 17), the heterogeneity results were obviously decreased after omitting some low-quality studies, which indicating the sensitivity is high and when interpreting the results and drawing conclusions should be careful. The funnel plot of the included studies is shown in Fig. 18. The points in the funnel plot were almost symmetrically distributed, indicating that the publication bias was not apparent and may affect the strength of the evidence.

## Discussion

The HTO, which appeared in the 1960s, was an accepted surgical treatment in medial compartment arthritis. With the progress of science and technology, the surgical methods of HTO are also developing, mainly including lateral closed wedge-shaped HTO, medial open wedge-shaped HTO. Studies have shown that HTO can effectively improve the biomechanical environment in the knee joint of patients with knee osteoarthritis, so as to reduce pain and improve knee function [41, 42].

PFO is a simple, trauma-minimized and effective procedure that enables patients to perform rehabilitation exercises and bear weight at earlier postoperative stage, which is widely used in recent decades [43]. PFO is based on the “non-uniform settlement” theory proposed by Zhang et al. [10, 11]. Early knee osteoarthritis is mostly manifested in the inward movement of the lower limb force line during weight bearing, resulting in the increase of local stress in the medial compartment of the knee joint and the narrowing of the medial joint space, resulting in pain and knee varus. According to the theory of uneven settlement, because the medial tibial plateau bears 2/3 of the body mass, while the lateral platform bears relatively less weight and has the support of fibula, it is not easy to collapse. Therefore, the tibia will be unbalanced in the process of human aging and osteoporosis, which shows that the settlement rate of the medial platform is significantly faster than that of the lateral platform, and finally, the genu varus intensifies, and the soft tissue around the knee gradually loses its balance and pulls the periosteum, resulting in joint pain, limited activity and deterioration of the disease. At this time, if the pressure on the inner side of the knee joint can be transferred to the outside to reduce the overload on the inner joint surface of the knee joint, the symptoms of the patient can be relieved to a great extent. The PFO proposed based on the theory of uneven settlement of the knee joint reduces the supporting force of the fibula on the lateral tibial platform by cutting off part of the bone at the proximal fibula and transfers part of the pressure to the outside, and with the outward movement of the knee joint load, the patient's lower limb force line can be recovered to avoid the aggravation of knee varus, so as to alleviate the patient's knee joint pain and improve the dysfunction. Mo et al. [44] used the three-dimensional finite element method to analyze the biomechanical changes of the tibial plateau caused by the PFO and found that there were significant changes in the stress on the tibial plateau before and after the PFO. Compared with before operation, the stress value of the medial tibial plateau decreased after simulated PFO, while the stress value of the lateral tibial plateau increased, indicating that high fibular osteotomy is indeed helpful to reduce the pressure on the medial tibial plateau, which is consistent with the results reported in clinical case studies.

### Summary of main results

Because both PFO and HTO are suitable for varus KOA, and both adopt the principle of changing the internal and external stress of the tibial plateau to adjust the varus deformity of the knee, there is a dispute about the choice of the two methods in clinic. Many clinicians have compared and reported the advantages and disadvantages and curative effects of the two methods, but due to the small sample size and other factors, the conclusions are often lack of persuasion. This paper expands the sample size of these clinical studies through meta-analysis to increase the reliability of the conclusions. Through the retrieval and screening of multiple databases, this paper makes a meta-analysis of 23 clinical studies of PFO compared with HTO, a total of 1460 patients with KOA. The heterogeneity analysis of most outcome indicators shows that there is great heterogeneity among the included studies. In order to increase the reliability of meta-analysis, the sensitivity analysis of highly heterogeneous indexes was carried out by eliminating some literature deviating from the forest map, and then, meta-analysis was carried out again. There was no significant difference for VAS and HSS, indicating that the two surgical methods can achieve the same short-term effect in the treatment of KOA. However, compared with the HTO group, the PFO group has significantly less average operation time, intraoperative bleeding and hospital stay, and the incidence of postoperative complications is also lower. The main reason may be that the HTO group needs to place internal fixation in addition to osteotomy, and the process is more complex, which increases the operation time and intraoperative bleeding, this leads to greater surgical trauma, increased risk of postoperative complications and longer time for weight bearing walking, which increases the length of hospital stay.

The complications in ten included studies also should be discussed. On the whole, 21 (7.7%) complications under PFO surgery were reported and 32 (11%) complications under HTO surgery were reported in 10 included studies [18, 19, 22, 26–28, 32, 35, 37, 38], which showed that PFO treatment has the lower complication rate than HTO treatment. The major complications reported after PFO surgery included superficial peroneal nerve traction injury, neurovascular injury, infection, knee instability, delayed healing. The major complications after HTO surgery included superficial peroneal nerve traction injury, neurovascular injury, patella baja, infection, numbness, knee instability, delayed healing, lower extremity deep venous thrombosis, pulmonary embolism, hinge fracture,recurrence deformity [18, 19, 22, 26–28, 32, 35, 37, 38].

### Limitations of the study

Through the comprehensive analysis of the included literature, it is found that the clinical studies included at present mainly have the following problems: 1. Too few subjects were included in some literatures, and the calculation basis of sample size was not presented; 2. Not all study outcome indicators have the same time point, and the time point of some study outcome indicators is not clear; 3. Most studies have a short follow-up time, so it is impossible to compare the medium- and long-term efficacy. The above problems can provide reference for the design of relevant clinical studies in the future. More large-sample, multicenter, high-quality, randomized controlled trials are needed to verify the outcomes of this meta-analysis.

## Conclusions

In conclusion, PFO and HTO have the same short-term efficacy in the treatment of KOA, but PFO can reduce the operation time, intraoperative bleeding, hospital stay and postoperative complications, which has certain advantages. Clinically, for patients with many complications and poor surgical tolerance, PFO can be preferred. In view of the heterogeneity and different follow-up time, whether these conclusions are applicable should be further determined in future studies [45–47].

## Data Availability

The present study was a review of previous published literatures.

## Abbreviations

KOA: Knee osteoarthritis
PFO: Proximal fibular osteotomy
HTO: High tibial osteotomy
TKA: Total knee arthroplasty
RCT: Randomized controlled trial
VAS: Visual analogue scale
KSS: Knee Society Score
HSS: Hospital for Special Surgery Knee Score
AKS: American Knee Society Score
WOMAC: Western Ontario and McMaster University of Orthopedic Index
FTA: Femur–tibia angle
CNKI: China National Knowledge Infrastructure
MD: Mean difference
CI: Confidence interval
OR: Odds ratio
